# *Schisandra chinensis* Pomace Attenuates Scopolamine-Induced Cholinergic Dysfunction Associated with Changes in BDNF and JNK Signaling

**DOI:** 10.3390/cimb48040390

**Published:** 2026-04-10

**Authors:** Ji Hye Yoon, Sung Ho Lim, In-Seo Lee, You Kyung Jang, Soeun J. Park, Song Ju Lee, Sangeun Im, Ji-Ho Park, Hyunwoo Park, Sungho Maeng, Jihwan Shin

**Affiliations:** 1 College of East-West Medical Science, Kyung Hee University, Yongin 17104, Republic of Korea; 2 Gyeonggi R&DB Center, 105, Gwanggyo-ro, Yeongtong-ro, Suwon-si 16229, Republic of Korea; 3 Epigenix Innovation, Niceville, FL 32578, USA; 4 Health Park Co., Ltd., Seoul 02447, Republic of Korea; 5 College of Pharmacy, Dongduk Women’s University, Seoul 02707, Republic of Korea

**Keywords:** *Schisandra chinensis* pomace, dietary by-product, synaptic plasticity, BDNF, JNK

## Abstract

Cholinergic dysfunction and impaired synaptic plasticity are key mechanisms underlying cognitive decline in neurodegenerative conditions, including Alzheimer’s disease (AD). *Schisandra chinensis* pomace (SSP), a by-product of fruit processing, contains bioactive lignans and polyphenols with reported neuroprotective properties; however, its effects under cholinergic dysfunction have not been systematically investigated. In this study, the effects of SSP on scopolamine-induced cognitive impairment were evaluated using ex vivo electrophysiological and in vivo behavioral approaches. Multi-electrode array recordings demonstrated that SSP at 0.1 mg/mL significantly restored scopolamine-suppressed hippocampal long-term potentiation (LTP), whereas a higher concentration (1.0 mg/mL) did not restore hippocampal synaptic potentiation. In vivo, C57BL/6N mice received oral SSP (50 or 100 mg/kg/day) for six weeks, with scopolamine administered during the final three weeks. SSP at 50 mg/kg prevented scopolamine-induced body weight loss, attenuated hyperlocomotor activity, and significantly improved memory retention, as evidenced by enhanced performance in the passive avoidance and Morris water maze tests. Furthermore, SSP restored hippocampal brain-derived neurotrophic factor (BDNF) expression and reduced the p-JNK/JNK ratio, indicating modulation of neurotrophic and stress-responsive signaling pathways. Collectively, these findings suggest that SSP attenuates scopolamine-induced cholinergic dysfunction, accompanied by improved hippocampal synaptic plasticity and changes in BDNF and JNK signaling. These results support the potential of SSP as a neuroactive botanical resource under cholinergic challenge.

## 1. Introduction

Neurodegenerative disorders are a major cause of cognitive decline worldwide. With the rapid growth of the aging population, the global economic and healthcare burden of AD continues to rise [1]. Pathological hallmarks of AD include extracellular deposition of β-amyloid (Aβ) plaques, intracellular neurofibrillary tangles formed by hyperphosphorylated tau, and widespread neuronal loss, which collectively lead to synaptic dysfunction and hippocampal atrophy, disrupting neural circuits essential for learning and memory [2,3].

Oxidative stress, neuroinflammation, mitochondrial dysfunction, and metabolic disturbances such as insulin resistance are increasingly recognized as important contributors to AD pathogenesis [4,5,6,7]. This multifactorial etiology complicates therapeutic development and highlights the importance of exploring multi-target biological strategies. Currently available treatments provide only symptomatic relief and fail to halt disease progression [8]. Natural products have attracted increasing attention as potential therapeutic candidates due to their multi-target biological effects, including modulation of oxidative stress, neuroinflammation, and synaptic signaling pathways [8].

Scopolamine, a non-selective muscarinic acetylcholine receptor antagonist, is widely used to induce transient cognitive impairment in rodents. Cholinergic dysfunction is a central feature of cognitive impairment and plays a critical role in learning and memory deficits. Although it does not replicate the full spectrum of AD pathology, this model primarily reflects cholinergic dysfunction and serves as a robust preclinical platform for evaluating candidate therapeutics [9,10]. While useful for assessing cholinergic-related cognitive impairment, this model does not recapitulate progressive neurodegenerative pathology associated with disorders such as Alzheimer’s disease. Therefore, the present study focuses on cholinergic dysfunction-related cognitive impairment rather than directly modeling Alzheimer’s disease-specific neuropathology.

BDNF plays a critical role in synaptic plasticity and memory formation, whereas c-Jun N-terminal kinase (JNK) signaling is involved in stress-related neuronal responses and synaptic regulation, and their dysregulation has been implicated in cognitive dysfunction. SSP, a by-product derived from the fruit of *S. chinensis*, has been associated with hepatoprotective, adaptogenic, and anti-fatigue properties [11]. Beyond these traditional uses, recent studies using *S. chinensis* fruit extracts have demonstrated improvements in memory, reductions in amyloid-β accumulation, and enhancement of cholinergic transmission in experimental models of cognitive impairment [12,13].

While most previous studies have focused on fruit extracts, SSP—a pomace fraction consisting of peel, pulp, and seeds—retains substantial levels of lignans, polyphenols, and flavonoids with potential biological activity [14,15,16]. Fruit pomace is often discarded despite containing bioactive compounds, representing an underutilized resource. From a sustainability perspective, the valorization of such by-products offers a cost-effective and resource-efficient strategy for identifying functional bioactive materials.

To our knowledge, the neurocognitive effects of *Schisandra chinensis* pomace have not been previously investigated. Therefore, this study focuses on SSP as a bioactive by-product rather than a conventional plant extract. These characteristics provide a rationale for investigating SSP in models of cholinergic dysfunction associated with cognitive impairment [14,15,16].

In this study, we examined whether SSP could attenuate scopolamine-induced cholinergic dysfunction and associated cognitive deficits. We assessed its effects on hippocampal synaptic plasticity using ex vivo multi-electrode array recordings, evaluated behavioral outcomes in memory-related tasks, and analyzed hippocampal expression of BDNF and JNK to elucidate the underlying mechanisms. These findings aim to characterize the effects of SSP on synaptic plasticity and behavioral outcomes in a scopolamine-induced model of cholinergic dysfunction.

## 2. Materials and Methods

### 2.1. Reagents and Materials

A standardized SSP extract, chemically characterized by HPLC analysis, was provided by QBM Co., Ltd. (Seoul, Republic of Korea). The pomace, composed of peel, pulp, and seeds remaining after fruit processing, was subjected to aqueous ethanol extraction and subsequently lyophilized into powder form, as described previously [14,15,16]. To characterize a representative bioactive component of SSP, schisandrin was quantified by HPLC using a C18 column with UV detection at 254 nm, and its identity was confirmed by comparison with an authentic reference standard. Schisandrin was selected as a representative marker compound because it is one of the major lignans present in *S. chinensis* and is commonly used as a quality control indicator for Schisandra-derived extracts. Quantitative analysis was conducted by a KOLAS-accredited analytical laboratory (Korea Health Functional Food Association). The chromatographic profile and certificate of analysis are provided in the Supplementary Materials (Figure S1 and Table S1).

### 2.2. Animals and Experimental Design

**Figure 1:**
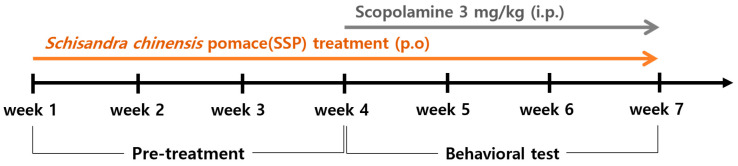
Experimental schedule for SSP administration. Eight-week-old female C57BL/6N mice were randomly assigned to five groups (n = 8/group): vehicle control (CTR; distilled water, p.o. + saline, i.p.), scopolamine control (SCO; distilled water, p.o. + scopolamine 3 mg/kg, i.p.), donepezil plus scopolamine (DNP; donepezil 3 mg/kg, p.o. + scopolamine 3 mg/kg, i.p.), SSP 50 mg/kg plus scopolamine (SSP50, p.o. + scopolamine 3 mg/kg, i.p.), and SSP 100 mg/kg plus scopolamine (SSP100, p.o. + scopolamine 3 mg/kg, i.p.). SSP and donepezil were administered once daily for six weeks. Scopolamine was injected intraperitoneally once daily during weeks 4–6, 30 min before behavioral testing. The timeline depicts adaptation, treatment, behavioral assessment, and tissue collection.

C57BL/6N mice (female, 8 weeks old, 18–20 g) were purchased from Daehan Bio Link Co., Ltd. (Eumseong, Republic of Korea) and maintained under standard laboratory conditions (23 ± 1 °C, 55 ± 10% humidity, 12:12 h light/dark cycle) with free access to food and water. Female mice were used in this study to maintain experimental consistency and to minimize variability in behavioral and electrophysiological assessments under the present experimental conditions. The allocation of animals to experimental groups was performed using a random assignment procedure to minimize potential selection bias. Animals were labeled with identification numbers prior to group allocation and were then randomly assigned to each experimental group. Mice were randomly assigned to five groups (n = 8 per group): (1) CTR: Vehicle control (distilled water p.o. + saline i.p.), (2) SCO: Scopolamine control (distilled water p.o. + scopolamine 3 mg/kg i.p.), (3) DNP: Donepezil (3 mg/kg p.o. + scopolamine 3 mg/kg i.p.), (4) SSP50: SSP 50 mg/kg-treated group (SSP 50 mg/kg p.o. + scopolamine 3 mg/kg i.p.), (5) SSP100: SSP 100 mg/kg-treated group (SSP 100 mg/kg p.o. + scopolamine 3 mg/kg i.p.). SSP or donepezil was administered orally once daily for six weeks. Scopolamine was injected intraperitoneally once daily during weeks 4–6 of the treatment period, 30 min before behavioral testing, whereas donepezil was administered orally once daily throughout the six-week treatment period. All behavioral experiments were performed between 09:00 and 17:00 (Figure 1). The inclusion criteria for the study were healthy female C57BL/6N mice of the specified age and body weight range. No predefined exclusion criteria were established prior to the experiments. During the course of the study, no animals were excluded and no mortality occurred.

All experimental procedures were approved by the Institutional Animal Care and Use Committee (IACUC) of Kyung Hee University (Approval No. KHGASP-22-552) and performed in accordance with the NIH Guide for the Care and Use of Laboratory Animals [17].

### 2.3. Electrophysiological Recording in Organotypic Hippocampal Slices

Organotypic hippocampal slice cultures (OHSCs) were prepared from Sprague–Dawley rats (postnatal day 7), a well-established preparation for stable long-term electrophysiological recordings [18]. Brains were removed, and hippocampi were isolated and sectioned into 350-μm slices using a McIlwain tissue chopper. Slices were placed on 0.4-μm pore membrane inserts (Millicell-CM, Millipore, Billerica, MA, USA) and maintained in 6-well plates with culture medium containing 50% MEM, 25% HBSS, 25% horse serum, 20 mM HEPES, 6 g/L glucose, 1 mM L-glutamine, and 1% penicillin–streptomycin at 36 °C in a 5% CO_2_ atmosphere. The medium was changed every 2–3 days, and slices were used after 14 days in vitro (DIV 14). Rat-derived organotypic hippocampal slices were selected because of their well-established stability and reproducibility in long-term LTP recordings.

For MEA recordings, slices were placed in polyethylenimine-coated MEA chambers (60MEA200/30iR, Multi Channel Systems, Reutlingen, Germany) and continuously perfused with artificial cerebrospinal fluid (aCSF) at 3 mL/min. Long-term potentiation (LTP) was induced by theta-burst stimulation (TBS; three trains of 100 Hz stimulation for 1 s each, delivered at 5 min intervals) applied to the Schaffer collateral pathway of the CA1 region [19]. Field excitatory postsynaptic potentials (fEPSPs) were recorded for 10 min before TBS and monitored for 95 min after stimulation. Drug treatment groups were: Control (aCSF), Scopolamine (300 μM), Scopolamine + SSP 0.1 mg/mL, and Scopolamine + SSP 1.0 mg/mL. For quantification, the mean slope of fEPSPs recorded 30–40 min post-TBS was normalized to the average baseline slope recorded during the 10 min preceding TBS.

### 2.4. Behavioral Assessments

Dose selection was guided by electrophysiological findings and preliminary behavioral results obtained from the passive avoidance test. Behavioral assessments and subsequent data analyses were conducted by investigators blinded to the treatment groups to reduce potential experimental bias. Ex vivo hippocampal recordings indicated a dose-dependent modulation of scopolamine-impaired LTP by SSP, and these observations were used to guide the selection of in vivo dosing ranges.

In preliminary behavioral screening using the passive avoidance test, memory performance was improved at moderate doses compared with higher doses, suggesting reduced efficacy at excessive concentrations. Based on these results, 100 mg/kg was selected as the upper effective dose, and 50 mg/kg, representing half of this dose, was chosen to evaluate efficacy in the lower range. This design allowed evaluation of stable effects at a moderate dose while also confirming the dose–response relationship, consistent with previous reports on Schisandra extract tolerability [12].

#### 2.4.1. Open Field Test (OFT)

The OFT was used to assess locomotor activity and anxiety-like behavior. Mice were placed individually in a Plexiglas arena (50 × 50 × 40 cm) illuminated at 20 lux and allowed to explore freely for 30 min. Total distance traveled and the percentage of time spent in the center zone were recorded using SMART v3.0 software (Panlab, Barcelona, Spain) [17].

#### 2.4.2. Passive Avoidance Test (PA)

A step-through passive avoidance apparatus (Scitech Korea, Seoul, Republic of Korea) was used to measure aversive memory. On the training day, mice received a 0.3 mA foot shock upon entering the dark compartment. Step-through latency was recorded immediately after training (0 h) and at 24 and 72 h during retention tests (cutoff: 300 s), conducted without foot shock [18].

#### 2.4.3. Morris Water Maze Test (MWM)

Spatial learning and memory were assessed using a circular pool (diameter: 180 cm) filled with opaque water maintained at 22 ± 2 °C. Mice were trained to locate a hidden platform with four trials per day for five consecutive days. Escape latency was measured in each trial. On day 6 (24 h after the final training session), a 90 s probe trial was performed with the platform removed. The number of crossings over the former platform location within the target quadrant was measured as an index of memory retention [19].

### 2.5. Western Blotting

After behavioral testing, mice were euthanized and hippocampal tissues were collected. Samples were homogenized in RIPA buffer containing phosphatase and protease inhibitors and centrifuged at 13,000 rpm for 15 min at 4 °C. Protein concentration was determined using the BCA assay (Thermo Fisher Scientific, Waltham, MA, USA). Equal amounts of protein (10 μg) were separated by SDS–PAGE, transferred to PVDF membranes, and probed with primary antibodies against brain-derived neurotrophic factor (BDNF), phospho-JNK, total JNK, and β-actin. After incubation with HRP-conjugated secondary antibodies, bands were visualized with enhanced chemiluminescence (ECL) and quantified using ImageJ software (version 1.54g, NIH, Bethesda, MD, USA) [20].

### 2.6. Statistical Analysis

Data are presented as mean ± SEM. Statistical analyses were performed using IBM SPSS Statistics version 29 (IBM Corp., Armonk, NY, USA) [21]. Normality and variance assumptions were assessed prior to analysis. Normally distributed data were analyzed using one-way ANOVA followed by Fisher’s LSD post hoc test, whereas non-parametric data were analyzed using the Kruskal–Wallis test with appropriate post hoc comparisons. Repeated-measures ANOVA was applied for time-course data where applicable. Sample sizes were determined based on previous studies using similar experimental designs, and a formal power analysis was not performed. A value of *p* < 0.05 was considered statistically significant.

## 3. Results

### 3.1. Sample Characterization (HPLC Analysis)

**Figure 2:**
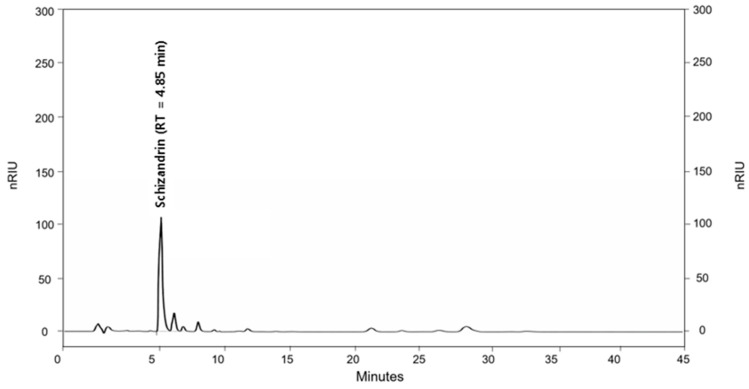
HPLC chromatographic profile of SSP extract. A representative chromatogram showing the schisandrin peak identified by comparison with the retention time of the authentic standard.

The chromatographic profile of SSP revealed a distinct peak corresponding to schisandrin, identified by comparison with the retention time of an authentic standard (Figure 2). Quantitative analysis revealed that SSP contained 46.68 mg/g of schisandrin, as determined by a KOLAS-accredited analytical laboratory. The major bioactive constituents of SSP are generally consistent; however, their relative contents may vary depending on factors such as harvest year, geographic origin, and extraction or processing conditions.

### 3.2. SSP Attenuates Scopolamine-Induced Impairment of Hippocampal LTP

**Figure 3:**
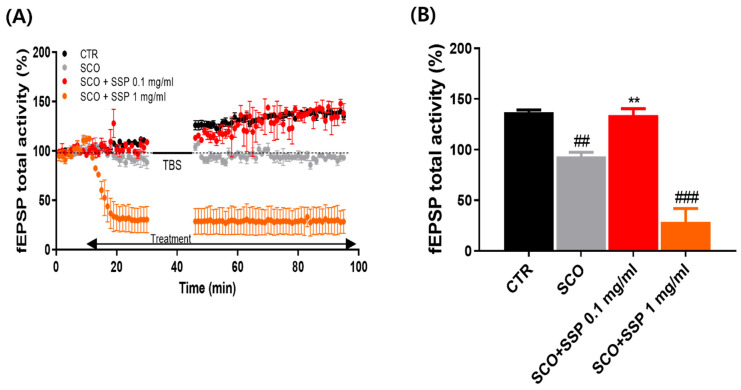
SSP attenuates scopolamine-induced impairment of hippocampal LTP in organotypic hippocampal slice cultures. (**A**) Time course of normalized fEPSP activity (% of baseline) following theta-burst stimulation (TBS) under four conditions: control (CTR), scopolamine (SCO, 300 μM), SCO + SSP 0.1 mg/mL, and SCO + SSP 1.0 mg/mL. Scopolamine attenuated LTP, whereas SSP (0.1 mg/mL) restored synaptic potentiation. SSP (1.0 mg/mL) did not improve LTP and reduced synaptic responses relative to the control group. (**B**) Quantification of normalized fEPSP activity recorded during the 30–40 min post-TBS interval. SSP (0.1 mg/mL) significantly increased LTP compared with the scopolamine group, whereas SSP (1.0 mg/mL) reduced synaptic potentiation relative to the control group. Data are presented as mean ± SEM (n = 3 per group). Statistical analysis was performed using one-way ANOVA followed by Fisher’s LSD post hoc test (## *p* < 0.01, ### *p* < 0.001 vs. CTR; ** *p* < 0.01 vs. SCO). CTR, control; SCO, scopolamine-treated group; SSP 0.1 mg/mL, SSP 1.0 mg/mL, SSP-treated groups.

Theta-burst stimulation (TBS) in control hippocampal slices induced significant long-term potentiation (LTP), evidenced by a sustained increase in normalized fEPSP activity relative to baseline. In contrast, slices treated with scopolamine (300 μM) showed significantly attenuated LTP compared with the control group. Co-treatment with SSP at 0.1 mg/mL significantly increased fEPSP activity compared with the scopolamine group (*p* < 0.01). In contrast, SSP at 1.0 mg/mL did not significantly improve LTP compared with the scopolamine group and showed reduced synaptic potentiation relative to control slices (*p* < 0.001 vs. CTR) (Figure 3).

### 3.3. SSP50 Attenuates Scopolamine-Induced Body Weight Loss

**Figure 4:**
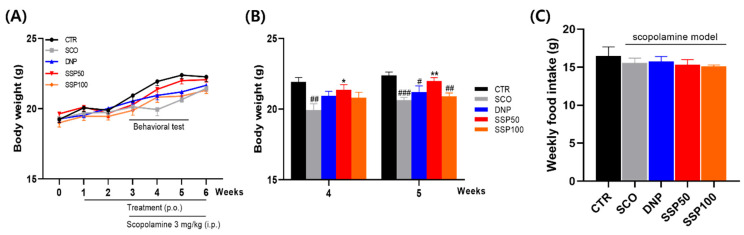
SSP attenuates scopolamine-induced body weight loss during the treatment period. (**A**) Weekly changes in body weight over the 6-week treatment period. (**B**) Between-group comparisons of body weight at weeks 4 and 5. (**C**) Weekly food intake during the experimental period. Data are presented as mean ± SEM (n = 8 per group). Body weight changes over time were analyzed using repeated-measures ANOVA. Between-group comparisons at specific time points were analyzed using the Kruskal–Wallis test with appropriate post hoc comparisons. Weekly food intake was analyzed using the Kruskal–Wallis test. (# *p* < 0.05, ## *p* < 0.01, ### *p* < 0.001 vs. CTR; * *p* < 0.05, ** *p* < 0.01 vs. SCO). CTR, vehicle control; SCO, scopolamine-treated group; DNP, donepezil-treated group; SSP50, SSP 50 mg/kg-treated group; SSP100, SSP 100 mg/kg-treated group.

Scopolamine treatment significantly reduced body weight compared with the control group, with a significant decrease observed at week 4 (*p* < 0.01 vs. CTR) and a more pronounced reduction at week 5 (*p* < 0.001 vs. CTR) (Figure 4A,B). SSP50 treatment significantly attenuated scopolamine-induced body weight loss, with significant differences observed at week 4 (*p* < 0.05 vs. SCO) and week 5 (*p* < 0.01 vs. SCO). At week 5, body weight in the SSP50 group was not significantly different from that in the control group. In contrast, SSP100 resulted in only partial recovery without a consistent effect across time points. Weekly food intake did not differ significantly among groups throughout the experimental period (Figure 4C), indicating that the observed changes in body weight were not attributable to alterations in dietary intake. These findings suggest that SSP50 attenuates scopolamine-induced body weight loss without significant alterations in food intake.

### 3.4. SSP Effects on Memory Retention and Spatial Learning in Behavioral Tests

**Figure 5:**
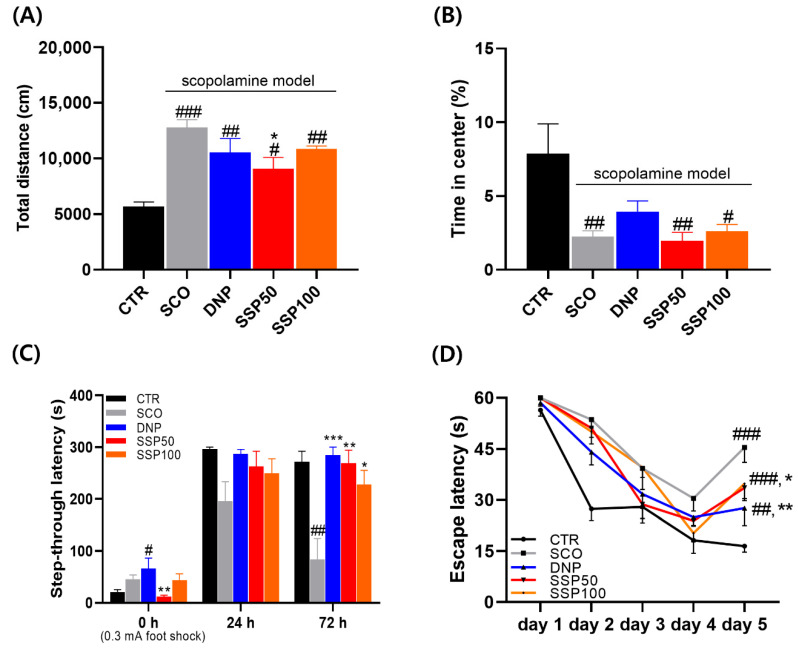
SSP effects on memory retention and spatial learning in behavioral tests. (**A**) Open field test. Total distance traveled. (**B**) Open field test. Percentage of time spent in the center zone. (**C**) Passive avoidance test. Step-through latency measured at 0, 24, and 72 h after training. (**D**) Morris water maze training. Escape latency across five consecutive training days. Data are presented as mean ± SEM (n = 8 per group). Open field and passive avoidance data were analyzed using the Kruskal–Wallis test followed by Bonferroni post hoc comparisons. Learning curves in the Morris water maze were analyzed using repeated-measures ANOVA, and between-group comparisons at specific time points were analyzed using the Kruskal–Wallis test with appropriate post hoc comparisons. (# *p* < 0.05, ## *p* < 0.01, ### *p* < 0.001 vs. CTR; * *p* < 0.05, ** *p* < 0.01, *** *p* < 0.001 vs. SCO). CTR, vehicle control; SCO, scopolamine-treated group; DNP, donepezil-treated group; SSP50, SSP 50 mg/kg–treated group; SSP100, SSP 100 mg/kg–treated group.

In the open field test, scopolamine-treated mice exhibited a significant increase in total distance traveled compared with the control group (*p* < 0.001), indicating enhanced locomotor activity. Donepezil and SSP100-treated groups also showed increased locomotor activity relative to the control. SSP50 significantly reduced total distance traveled compared with the scopolamine group (*p* < 0.05 vs. SCO), indicating attenuation of scopolamine-induced hyperactivity; however, locomotor activity remained significantly higher than that of the control group (*p* < 0.05 vs. CTR) (Figure 5A). Regarding anxiety-like behavior, scopolamine significantly reduced the percentage of time spent in the center zone compared with the control group (*p* < 0.05). Neither SSP50 nor SSP100 treatment significantly restored center-zone exploration compared with the scopolamine group, indicating that SSP did not significantly modulate anxiety-like behavior under the present experimental conditions (Figure 5B). In the passive avoidance test, scopolamine-treated mice showed a significant reduction in step-through latency at 72 h compared with the control group (*p* < 0.05), indicating impaired long-term memory retention. SSP50 significantly prolonged step-through latency compared with the scopolamine group (*p* < 0.05 vs. SCO), approaching values observed in the donepezil-treated group, whereas SSP100 exhibited a partial but non-significant effect (Figure 5C). In the Morris water maze, all experimental groups exhibited progressive reductions in escape latency across training days, indicating intact learning ability (*p* < 0.001 for the effect of time). However, scopolamine-treated mice displayed significantly prolonged escape latency on day 5 compared with the control group (*p* < 0.001). SSP50 significantly shortened escape latency relative to the scopolamine group (*p* < 0.05 vs. SCO), although performance did not fully recover to control levels, whereas SSP100 did not produce a statistically significant improvement (Figure 5D).

### 3.5. SSP Modulates BDNF Expression and JNK Activation in the Hippocampus

**Figure 6:**
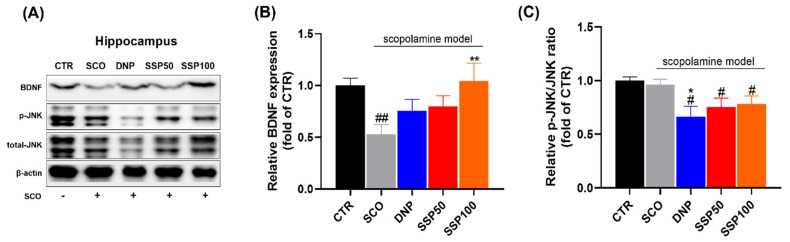
SSP modulates hippocampal BDNF expression and JNK signaling. (**A**) Representative Western blot images of BDNF, phosphorylated JNK (p-JNK), total JNK, and β-actin in hippocampal tissues. (**B**) Quantification of BDNF protein expression normalized to β-actin. (**C**) Quantification of the p-JNK/total JNK ratio. Data are presented as mean ± SEM (n = 6–8 per group). Statistical analysis was performed using the Kruskal–Wallis test with appropriate post hoc comparisons (# *p* < 0.05, ## *p* < 0.01 vs. CTR; * *p* < 0.05, ** *p* < 0.01 vs. SCO). CTR, vehicle control; SCO, scopolamine-treated group; DNP, donepezil-treated group; SSP50, SSP 50 mg/kg–treated group; SSP100, SSP 100 mg/kg–treated group.

Western blot analysis revealed that scopolamine significantly reduced hippocampal BDNF expression compared with the control group (*p* < 0.01). SSP100 significantly increased BDNF expression compared with the scopolamine group (*p* < 0.01), partially restoring BDNF levels toward those of the control group. The p-JNK/JNK ratio was significantly increased in the scopolamine group compared with the control group (*p* < 0.05). Donepezil, SSP50, and SSP100 significantly reduced the p-JNK/JNK ratio compared with the scopolamine group (*p* < 0.05). Total JNK expression did not differ significantly among groups (Figure 6).

## 4. Discussion

This study evaluated the effects of SSP, a fruit-processing by-product, in a scopolamine-induced model of cholinergic dysfunction. SSP improved hippocampal synaptic plasticity, behavioral performance, and signaling changes related to BDNF and JNK. These results support the view that bioactive constituents retained in the pomace fraction contribute to cognitive protection under cholinergic challenge.

Scopolamine-induced impairment of hippocampal LTP is widely used as an electrophysiological indicator of cholinergic-related memory dysfunction [9,10]. In the present study, scopolamine markedly reduced hippocampal LTP, supporting the validity of the model at the synaptic level.

SSP showed a bidirectional effect on synaptic plasticity. The lower concentration (0.1 mg/mL) restored LTP, whereas the higher concentration (1.0 mg/mL) failed to rescue synaptic potentiation and instead reduced synaptic responses. This pattern indicates a concentration-dependent effect of SSP on hippocampal function. One possible explanation is that, at higher concentrations, certain constituents may interfere with neuronal signaling and thereby limit synaptic enhancement.

SSP appears to operate within a relatively narrow effective range for synaptic plasticity. Schisandrin has been reported to support neuroprotection and synaptic function at appropriate doses [12], but SSP is a multi-component pomace extract containing lignans and seed-derived constituents [14,15,16]. The reduced efficacy observed at the higher concentration may therefore reflect interactions among multiple constituents rather than the action of a single compound. Further studies are needed to define the optimal dose range and identify the components responsible for this effect. These electrophysiological findings were further evaluated in behavioral paradigms.

The in vivo dose range was selected to test whether the concentration-dependent electrophysiological pattern would translate into behavioral outcomes. Preliminary passive avoidance findings, together with the ex vivo LTP results, supported the selection of 50 and 100 mg/kg for subsequent analyses. This design allowed comparison of an effective dose range with a less effective condition in vivo.

Scopolamine reduced body weight during the experimental period, consistent with previous reports of chronic anticholinergic exposure [9]. SSP50 attenuated this effect, whereas SSP100 showed only partial recovery. Because weekly food intake did not differ among groups, the body weight change was unlikely to be explained by altered feeding behavior. This finding suggests that SSP50 may have influenced physiological or metabolic processes disrupted by scopolamine.

In the open field test, scopolamine increased locomotor activity, consistent with previous reports in scopolamine-based cognitive impairment models [9,17]. SSP50 attenuated this hyperactivity, whereas SSP100 and donepezil showed less consistent effects. By contrast, SSP did not significantly reverse the reduction in center-zone exploration, indicating limited effects on anxiety-like behavior under the present conditions.

In the passive avoidance test, scopolamine impaired long-term aversive memory, reflected by reduced step-through latency at 72 h. SSP50 markedly improved retention, whereas SSP100 produced only a modest and non-significant effect. These results further support the greater efficacy of the lower dose.

In the Morris water maze, all groups showed reduced escape latency across training days, indicating preserved task acquisition [19]. However, scopolamine impaired spatial learning on the final training day. SSP50 partially restored performance, whereas SSP100 did not produce a significant improvement. Because neither SSP dose fully recovered target-quadrant preference in the probe test, the effect of SSP appears to have been stronger on learning performance than on memory consolidation.

This consistency between electrophysiological and behavioral findings strengthens the interpretation that SSP exerts dose-dependent effects across multiple outcome measures.

The weaker efficacy observed at the higher dose may reflect the complex composition of SSP, which contains multiple lignans and seed-derived constituents [14,15]. At elevated concentrations, some of these components may interfere with synaptic signaling and thereby reduce overall efficacy. This finding underscores the importance of dose optimization for multi-component botanical preparations.

To explore potential mechanisms underlying these effects, molecular analyses were conducted.

Oxidative stress and neuroinflammatory signaling are closely linked to scopolamine-induced cognitive dysfunction, but these markers were not directly assessed in the present study [22,23,24]. Instead, the study focused on synaptic plasticity, behavior, and BDNF/JNK-related signaling. Because BDNF contributes to synaptic plasticity and memory, whereas JNK is associated with stress-related neuronal responses [4,23,24], the observed molecular changes may be related to the electrophysiological and behavioral effects of SSP. However, these findings remain correlative, and pathway-specific studies will be required to establish causality.

**Figure 7:**
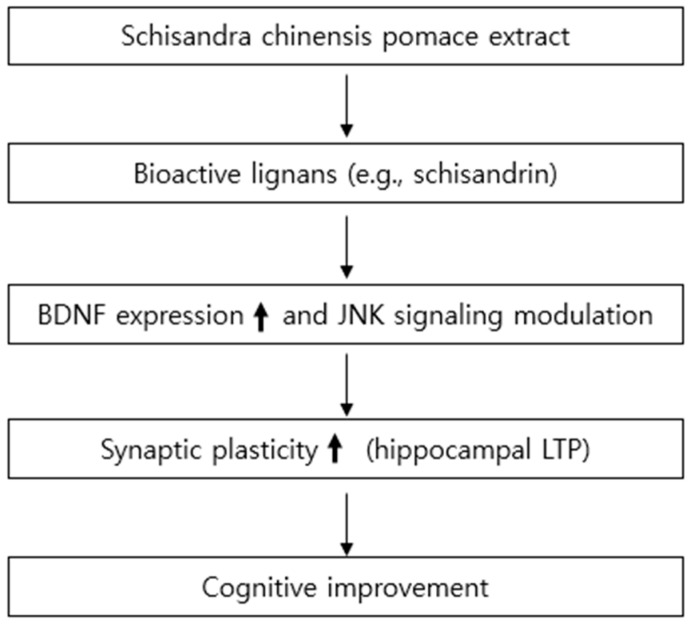
Proposed mechanism underlying the cognitive effects of SSP. Bioactive lignans present in SSP, including schisandrin, may be associated with changes in neurotrophic and stress-related signaling pathways such as BDNF and JNK. These molecular changes are associated with enhanced hippocampal synaptic plasticity, including long-term potentiation (LTP), which may contribute to improved cognitive performance in the scopolamine-induced model. Upward arrows (↑) indicate increased expression or activity.

Although the present molecular data do not establish causality, they support a working model linking SSP treatment to changes in BDNF/JNK-related signaling and synaptic plasticity. This proposed interpretation is summarized schematically in Figure 7.

SSP differentially modulated BDNF and JNK signaling across the tested dose range. SSP50 was more closely associated with reduced JNK activation, whereas SSP100 showed a stronger effect on BDNF expression. This divergence suggests that the two doses may engage partly distinct signaling profiles, with the lower dose showing better correspondence with LTP recovery and behavioral improvement.

Although SSP100 increased BDNF expression, this change was not accompanied by clear recovery of synaptic plasticity or behavioral performance. This mismatch suggests that BDNF upregulation alone may not be sufficient to restore cognitive function in the presence of other inhibitory influences.

Behavioral findings were broadly consistent with this interpretation. SSP50 improved passive avoidance and Morris water maze performance, whereas its effects on anxiety-like behavior were limited [17]. In addition, the protection against body weight loss occurred without a change in food intake, suggesting that this effect was not driven by altered caloric consumption.

Among the tested doses, SSP50 showed the most consistent profile across electrophysiological, behavioral, and molecular outcomes. This comparison highlights the importance of dose selection when evaluating complex botanical by-products.

The present study differs from earlier work by focusing on the pomace fraction rather than the fruit extract itself. Although S. chinensis extracts have shown neuroprotective effects in cognitive impairment models [12,13], SSP remains an underexplored by-product that retains lignans, polyphenols, and related phytochemicals [11,14,16]. These constituents may underlie the electrophysiological, behavioral, and molecular effects observed here. In addition, previous studies have reported antioxidant and stress-protective properties in Schisandra-derived extracts and by-products [4,14,22], which may be relevant to scopolamine-induced dysfunction. Further phytochemical profiling will be necessary to identify the specific constituents responsible for SSP activity. Overall, the current findings support the biological relevance of SSP as a functional by-product rather than an inert waste material.

More broadly, these findings support the value of agricultural by-products as sources of functional bioactive materials. Because pomace is often underutilized despite retaining biologically active phytochemicals [14,15,16], the activity observed for SSP supports the feasibility of repurposing food-processing residues for neurophysiological research.

Several limitations should be acknowledged. First, oxidative stress and inflammatory markers were not directly assessed. Second, the scopolamine model reflects acute cholinergic dysfunction and does not reproduce progressive neuropathological features such as amyloid or tau accumulation [2,8]. Third, only female mice were used, which may limit generalizability given known sex-dependent differences in stress responses, metabolism, and cognition. Future studies including both sexes will be needed to determine whether the effects of Schisandra chinensis pomace are sex-dependent. Finally, the present molecular findings remain correlative because functional validation was not performed.

The precise molecular targets of SSP and the interactions among its individual constituents remain unclear. Future studies using fractionation, receptor-level analyses, and disease-relevant chronic models will help define the mechanisms underlying SSP-mediated cognitive modulation.

In summary, Schisandra chinensis pomace modulated synaptic plasticity, behavior, and BDNF/JNK-related signaling in a scopolamine-induced model of cholinergic dysfunction. Across the tested conditions, SSP50 showed the most consistent efficacy. These findings support further investigation of bioactive compounds retained in fruit-processing by-products as neurophysiologically relevant functional materials.

## 5. Conclusions

This study suggests that SSP, a fruit-processing by-product, is associated with changes in cognitive performance and hippocampal synaptic plasticity in a scopolamine-induced model of cholinergic dysfunction. SSP, particularly at the lower effective dose, was associated with restoration of hippocampal long-term potentiation, improved memory performance in behavioral tasks, and dose-dependent modulation of brain-derived neurotrophic factor (BDNF) and c-Jun N-terminal kinase (JNK) signaling.

Within the tested dose range, SSP50 was associated with improved cognitive performance, as reflected by prolonged step-through latency in the passive avoidance test and reduced escape latency in the Morris water maze, whereas SSP100 showed limited or inconsistent efficacy. These behavioral outcomes were accompanied by reduced JNK activation at the lower dose, whereas the higher dose was associated with increased BDNF expression, indicating differential and pathway-selective molecular modulation across the tested dose range.

Collectively, the present findings suggest that bioactive compounds retained in SSP are associated with synaptic plasticity and cognitive performance under conditions of cholinergic dysfunction. The utilization of SSP also highlights the potential value of underexplored agricultural by-products as functional materials for neurophysiological research, aligning with principles of sustainability and resource valorization. Further studies are required to define the specific active constituents and to evaluate the effects of SSP in additional experimental models of cognitive dysfunction.

## Data Availability

The original contributions presented in this study are included in the article/Supplementary Materials. Further inquiries can be directed to the corresponding author.

## Supplementary Materials

The following supporting information can be downloaded at https://www.mdpi.com/article/10.3390/cimb48040390/s1.

## Abbreviations

The following abbreviations are used in this manuscript:

SSP	*Schisandra chinensis* pomace
SCO	Scopolamine
DNP	Donepezil
LTP	Long-term potentiation
BDNF	Brain-derived neurotrophic factor
JNK	c-Jun N-terminal kinase
p-JNK	Phosphorylated c-Jun N-terminal kinase
CTR	Control
SEM	Standard error of the mean
