# Supplementary material for: Schisandra chinensis Pomace Attenuates Scopolamine-Induced Cholinergic Dysfunction Associated with Changes in BDNF and JNK Signaling
Source: Curr Issues Mol Biol. 2026 Apr 10;48(4):390. doi: 10.3390/cimb48040390 (PMC13115013; doi:10.3390/cimb48040390)
Supplement: Supplementary file 1 [file cimb-48-00390-s001.zip › cimb-4204782-supplementary.pdf]

Supplementary Figure S1. Representative HPLC chromatogram of *Schisandra chinensis* pomace (SSP) extract.

The schisandrin peak was identified by comparison with the retention time of an authentic schisandrin standard. Quantitative analysis was conducted by a KOLAS-accredited analytical laboratory.

Data File : C:\projects\Jung eun sook\Schizandrin\Result\KHSI-A-128 (Offline).39 2021-12-24 11-01-46 (GMT +09-00).rslt\d2021120516-1^10 2021-12-23 20-26-21 (gmt +09-00).dat
Sample ID : D2021120516-1^10
Method : C:\projects\Jung eun sook\Schizandrin\Method\Schizandrin(The korea pharmacopoeia)-CAL.met
Acquired : 2021-12-23 8:27:35 (GMT +09:00)
User name : Jung eun sook (A\Jung eun sook)

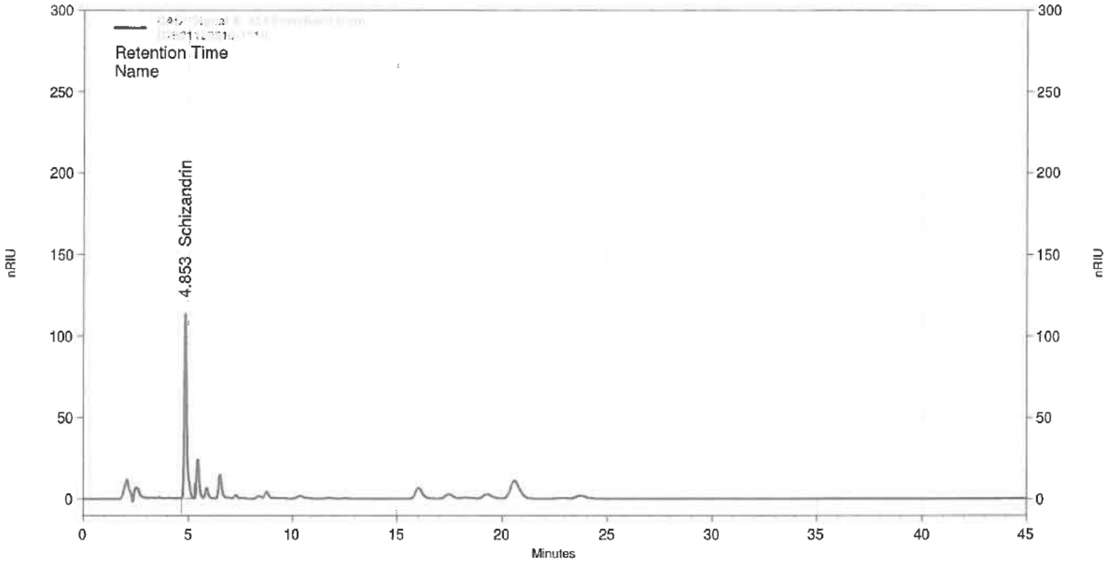

Channel A

DAD: Signal A, 254.0 nm/Bw:4.0 nm Results

Pk #	Name	Time	Area	Height	ESTD concentration
1	Schizandrin	4.853	122165195	15185318	54.2230

Supplementary Table S1. Schisandrin content in Schisandra chinensis pomace (SSP)

Measurement	Schisandrin content (mg/g)
1	46.64
2	46.15
3	47.20
Mean	46.68
